# Global trends and future directions in online learning for medical students during and after the COVID-19 pandemic: A bibliometric and visualization analysis

**DOI:** 10.1097/MD.0000000000035377

**Published:** 2023-12-15

**Authors:** Pei Zhang, Xiuyuan Li, Ying Pan, Haihun Zhai, Tian Li

**Affiliations:** a Department of Medicine-Education Coordination and Medical Education Research Center, Hebei Medical University, Shijiazhuang, China; b School of Basic Medicine, Fourth Military Medical University, Xi’an, China.

**Keywords:** bibliometric analysis, cite space, COVID-19, medical education, medical students, online learning

## Abstract

This study explores the evolution of online learning research in the context of medical education during and following the COVID-19 pandemic. It aims to understand the principal focus areas, and trends that have emerged in this rapidly evolving landscape. A total of 2751 publications related to online learning were retrieved from the Web of Science Core Collection (WoSCC) from 2020 to 2022. Bibliometric analysis and visualization techniques were employed to comprehensively examine the landscape of online learning research. Publications, co-cited references, and keyword co-occurrence were analyzed to identify patterns and trends in research focus and collaboration networks. The significant surge in research output reveals the academic community’s response to the pandemic. Various themes have emerged in online learning research, encompassing online teaching, flipped classrooms, mental health, and blended learning. The evolution trajectory of research has traversed 3 stages, reflecting a shift in research focus from immediate pandemic responses to more refined strategies and interdisciplinary perspectives. Keyword co-occurrence analysis was also conducted to show the effects of the COVID-19 pandemic on the research. The study underscores the global scholarly engagement, collaborative networks, and principal themes that have shaped the field. As medical education adapts to the shifting landscape, the trajectory of online learning research points toward increased learner autonomy, integration of advanced technologies, and interdisciplinary collaboration. This transformative shift promises to reshape medical education, equipping learners and educators with the tools needed to navigate the dynamic realm of modern healthcare education.

## 1. Introduction

Nowadays the COVID-19 pandemic is no longer a “global public health emergency,” but is defined as an established and sustained public health problem. However, it is undeniable that in the past 3 years, the pandemic has changed the world dramatically, including medical education. Since 2020, some of the most discussed topics in medical education academic have been the impact of the pandemic and the exploration of online learning practice. Although Covid is new, the online learning are not. Online learning had been expanding for 2 decades prior to the COVID-19 pandemic. Distance or asynchronous teaching and learning within higher education in the US, for example, began during the mid to late 1990s.^[1]^ It has further blossomed with the development of Massive Open Online Courses (MOOCs) beginning in 2012 – especially once some of the most prestigious universities in the US (and worldwide) embraced this technology and shift in learning methodology. However, higher education does not see e-learning as replacing traditional instructor-led training but as a complement to it, forming part of a blended-learning strategy. As COVID-19 has swept the world, face-to-face teaching and learning have been interrupted by the lockdown. Online learning has helped those medical schools to overcome established barriers overnight and make the most rapid online curricular shift in medical education’s history.^[2]^

Regarding the concept of online learning, this paper recommends Singh and Thurman’s definition of using the Internet to enhance students’ synchronous or asynchronous learning activities. Online learning relates to the concepts of e-learning, distance learning, remote learning, and tele-learning, which involve the use of technology to facilitate communication between teachers and students, regardless of the student’s physical or virtual location.^[3]^ As a sudden transition brought about by the COVID-19 pandemic, online learning can quickly explore and solve problems in response to impending challenges brought to medical education. A few published papers both domestically and internationally have employed bibliometric analysis to examine the impact of COVID-19 on online medical education. These publications predominantly focus on factors of influence,^[4]^ challenges and barriers,^[5]^ satisfaction of online medical education.^[6]^ However, there are few systematic reviews on the changes in online learning practices of medical students. This study reveals the research trends and current status of the publications which focused on COVID-19-related online learning through bibliometric and visualization analysis. It further provides important findings for the future research vision of this subject area.

## 2. Materials and methods

### 2.1. Data collection

The data were retrieved from the Web of Science Core Collection (WoSCC) to acquire publications focused on online learning. The search strategies were as follows: TS = (e-learning OR online learning OR distance learning OR tele learning OR remote learning) AND TS = (medical student) NOT TS = (veterinary). All searches were performed on a single day (October 1, 2022) to avoid bias produced by daily database renewal. The slice is 1 year. A total of 2751 records published from January 1, 2020, to September 30, 2022, were obtained for further study, including articles and reviews written in English. We obtained 2582 records after removing the duplicates, news, comments, editorials, and letters.

### 2.2. Software and methods

We chiefly used bibliometrics and visualization as the main methods for our research. Bibliometrics can reduce the distortion and bias caused by filtering subjective information by conducting comprehensive mining and research of basic literature. All the publications included were first exported in TEXT format and then imported into Cite Space (6.1.R3), which is the visualization software developed by Professor Chaomei Chen. Basic information, such as the yearly output and citation frequency was obtained from the WoSCC. Regarding the selection of time slices, a slice of 1 year was used because of the higher modularity value and the silhouette value of the clustering effect. Regarding the connection strength, cosine was used. Regarding the threshold, we selected the top 50 nodes at each time point. Moreover, the pruning used pathfinder and the merged network. By adjusting corresponding parameters, cluster analysis and timeline view were performed for keywords. The visualization functions of Cite Space, such as cooperative networks, co-cited documents, and keywords, were performed to explore emerging topics and future directions.^[4]^ It is important to note that we have effectively distinguished authors sharing the same abbreviation. In cases where authors have multiple affiliations, we have recorded the first one. Keywords with different expressions have been standardized to a single keyword. Furthermore, publications originating from Hong Kong, Macau, and Taiwan have been reclassified under China, while publications from England, Scotland, Northern Ireland, and Wales have been reclassified under the United Kingdom.

Some terms used in Cite Space need to be explained. In this study, different nodes in visualization represent different research subjects, such as “keyword.” Frequency is used to describe the number of nodes. The higher the frequency, the larger the circle of nodes. The purple ring outside the node represents the centrality; as the centrality increases, the amount of the core in the relationship also increases. Professor Chen believes that nodes with an intermediate centrality of > 0.1 play an important role in bridge connection.^[7]^ A line between nodes refers to a cooperative network; the thicker the lines, the closer the relationship; the same year is represented by the same color.

## 3. Results

### 3.1. Data analysis on publications

**Figure 1.**
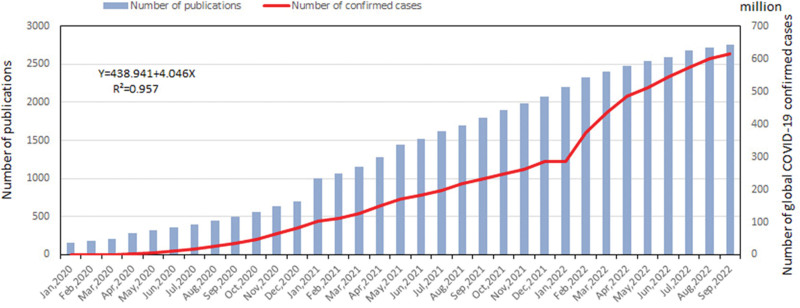
Number of global confirmed cases of COVID-19 and online learning publications.

Since the outbreak of the pandemic, 2751 studies on the topic have been published. Of these, 702 were published in 2020, 1377 in 2021, and 672 in 2022 from January to September. Figure 1 demonstrates an overall upward trend, especially at the beginning of 2021. The linear fitting of the studies shows a significant positive correlation (*R*^2^ = 0.957**) between the number of global COVID-19 confirmed cases (red curve) as reported by the WHO (https://covid19.who.int/)^[8]^ and the number of publications (blue column) of the WoSCC. Statistical analysis was performed by using one-way analysis of variance (ANOVA). An ANOVA are reported as F(df), *P* value, where df represents degrees of freedom (ANOVA (inter-group), F = 356.945, *P* < .001, df = 1). We can see that the 2 curves show almost the same growth tendency, which demonstrates the efforts made by the medical education academia to rapidly respond to the pandemic.

### 3.2. Cooperation relationship analysis

We mapped a cooperative relationship network for 119 countries, 252 institutions, and authors of 170 publications using Cite Space (6.1. R3). An analysis of the distribution of publications (Table 1) identified the top 10 productive countries, institutions, and authors. These findings can help reveal collaboration patterns, emphasize pivotal factors facilitating cooperation, foster global collaboration, and aid decision-making for future partnerships and resource allocation.

**Table 1:** Top 10 countries, institutions, and authors with the highest number of publications.

Rank	1	2	3	4	5	6	7	8	9	10
Country										
Name	USA	UK	GERMANY	CHINA	INDIA	AUSTRALIA	CANADA	SAUDI ARABIA	PAKISTAN	IRAN
Count	721	266	204	213	136	136	130	109	66	56
Institution										
Name	University Calif San Francisco	University of Toronto	Harvard Medical School	University of Michigan	Imperial College London	University of North Carolina	University of Sydney	University of Colorado	University of Stanford	University of Washington
Location	USA	AUSTRALIA	USA	USA	UK	USA	AUSTRALIA	USA	USA	USA
Count	36	33	33	29	27	26	25	24	22	21
Author										
Name	Yunhe Wang	Lee, Justin S	Xin Zhang	Yanfen Zhang	Xiaoming Liu	Kelly M. Harrell	Royer, Danielle	Martindale, Jim	Harmon, Derek	Jun Wang
Institution of Author	Peking University	CDC COVID-19 Response Team	University of Hong Kong	The Second Affiliated Hospital of Harbin Medical University	Wuhan University of Science and Technology	Virginia Commonwealth University School of Medicine	University of Colorado Anschutz Medical Campus	University of Virginia	University of California, San Francisco	The First Affiliated Hospital of Chongqing Medical University
Location	CHINA	USA	CHINA	CHINA	CHINA	USA	USA	USA	USA	CHINA
Count	15	13	12	12	12	12	11	10	10	10

#### 3.2.1. Co-institution analysis.

**Figure 2.**
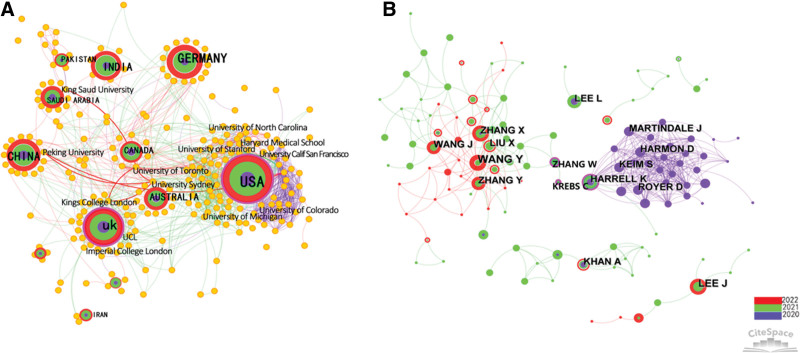
Cooperative relationship between countries, institutions (A), and authors (B) for online learning.

Institutions with a high frequency and centrality have also contributed to the promotion of global online learning research. Universities, the main organization for online learning research, have done more work than hospitals and research institutions. Figure 2A shows a close cooperative relationship among 252 institutions. Given the long history of online learning in the USA, 7 of the 10 most productive organizations worldwide are from the country. The University of California, San Francisco, had the highest number of publications (count = 36). Eight institutions had a centrality of > 0.1. Boston University (0.13), Brown University (0.12), and Johns Hopkins University (0.10) of the United States; McGill University (0.13) and Sydney University (0.12) of Australia; Kings College of London of the United Kingdom; Peking University (0.12) of China; and King Abdulaziz University (0.12) of Saudi Arabia (the nodes are linked by red lines) played an important role in establishing close cooperation between their countries.

#### 3.2.2. Co-country (Area) analysis.

The cooperative relationship between institutions also implies the corresponding relationship between the respective countries to some extent, as institutions belong to countries. Figure 2A shows the network map between 119 countries (areas). The United States has made the most outstanding contributions in the field of online learning in response to the COVID-19 pandemic, as evident from the 2 indicators of maximum publications (721) and highest centrality (0.3). The country in the second place is the United Kingdom, with 226 publications and 0.11 centrality. Both the US and UK have purple outer rings, which imply that these countries occupy the core position (Table 1). Next is Germany (count = 204, centrality = 0.04). China, ranking fourth in terms of publications (213) and 20th in terms of centrality (0.03), has also made great strides in online learning. Although some underdeveloped countries also appeared in the list, they have low centrality and they still need to improve the depth and breadth of their research by strengthening their communication and cooperation.

#### 3.2.3. Coauthor analysis.

Although the current work considered 241 authors based on the results of Cite Space analysis, only 170 of them met the minimum publication threshold (*N*_i_ ≥ 2.90), which was determined using Price’s formula $\left(N_{i}=0.749\sqrt{N_{m}}\right)$. Here, *N*_m_ refers to the number of papers issued by the most productive author. Figure 2B depicts the author cooperation relationship map, which shows a decentralized partnership of online learning. An obvious close cooperative network is composed of the authors with the highest number of publications. Most of these authors (more than 30 researchers) are from the universities based in the United States. Another obvious author cooperation network is noticed in China. It is clear from Figure 2 that 3 authors – Kelly M. Harrell, Wenlu Zhang, and Claudia Krebs – with a centrality of 0.13 have played an important intermediary role between China and the US.

### 3.3. Co-cited reference analysis

**Figure 3.**
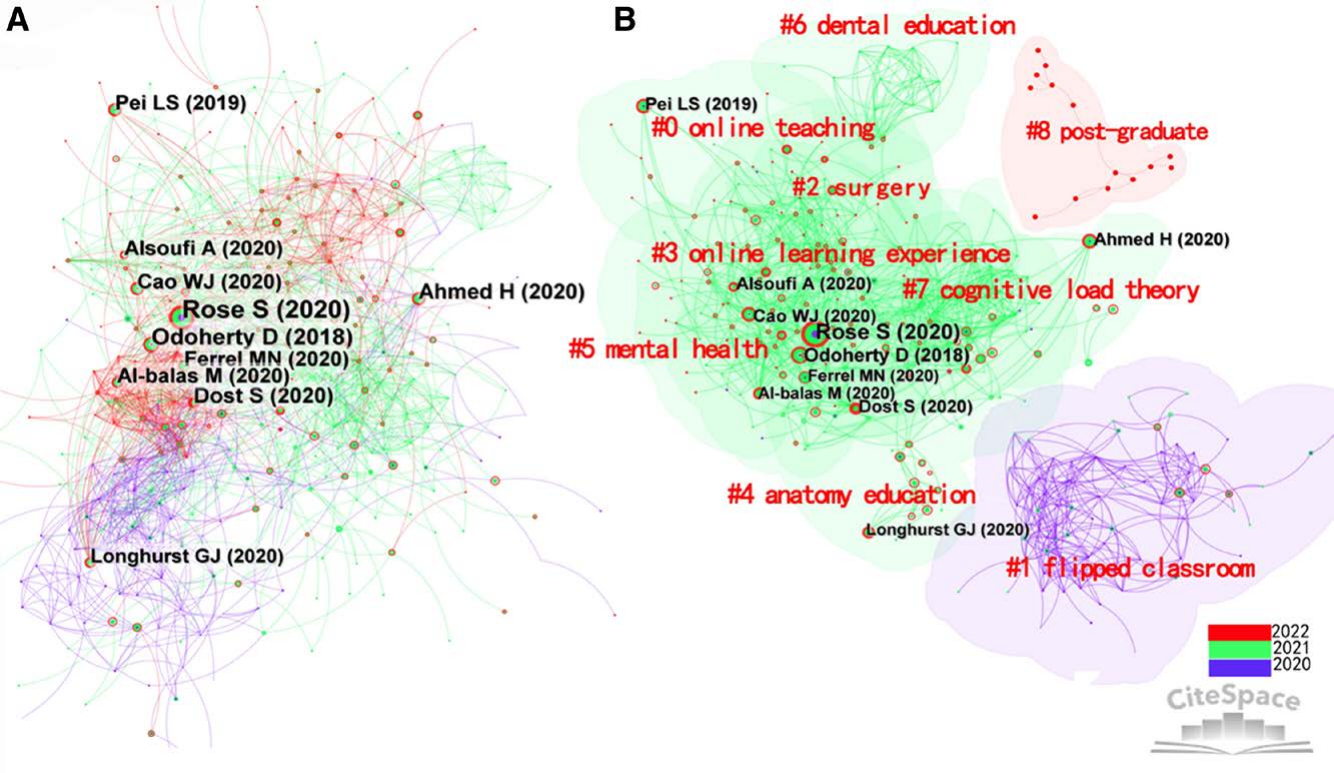
Co-cited references (A) and the cluster of co-cited references (B) of online learning.

Co-cited references of Cite Space refer to the phenomenon when 2 references are cited by the same document, making significant contributions to the evolution of knowledge. Figure 3A is a map of 2565 co-cited references cited by the authors. All of the top 10 co-cited references (Table 2) were published before 2020, such as Rose,^[9]^ Dost,^[10]^ and Ahmed.^[11]^ This shows that studies published before 2020 received wide attention after the outbreak of COVID-19, for example, the second most cited article written by O’Doherty (2018)^[12]^ and the fourth cited article written by Pei (2019).^[13]^ Note that the authors in the co-cited reference list are different from those in the coauthor list. Hence, a most productive author may not necessarily signify high quality of the study (or the quality of the journal).

**Table 2:** Top 10 co-cited references of online learning.

Rank	Year	Title	Author	Journal	Co-citation
1	2020	Medical Student Education in the Time of COVID-19	Rose S	JAMA-Journal of the American Medical Association	172
2	2018	Barriers and solutions to online learning in medical education - an integrative review	ODoherty D	BMC Medical Education	98
3	2020	Perceptions of medical students towards online teaching during the COVID-19 pandemic: a national cross-sectional survey of 2721 UK medical students	Dost S	BMJ OPEN	72
4	2019	Does online learning work better than offline learning in undergraduate medical education? A systematic review and meta-analysis	Pei LS	Medical Education Online	68
5	2020	COVID-19 and medical education	Ahmed H	LANCET Infectious Diseases	65
6	2020	Impact of the COVID-19 pandemic on medical education: Medical students’ knowledge, attitudes, and practices regarding electronic learning	Alsoufi A	PLOS ONE	63
7	2020	The psychological impact of the COVID-19 epidemic on college students in China	Cao WJ	Psychiatry Research	59
8	2020	Distance learning in clinical medical education amid COVID-19 pandemic in Jordan: current situation, challenges, and perspectives	Al-Balas M	BMC Medical Education	58
9	2020	The Impact of COVID-19 on Medical Education	Ferrel MN	Cureus Journal of Medical Science	58
10	2020	Strength, weakness, opportunity, threat analysis of the adaptations to anatomical education in the United Kingdom and Republic of Ireland in response to the Covid-19 pandemic	Longhurst GJ	Anatomical Sciences Education	52

The cited reference clustering label can be used to depict the core academic ideas and research frontiers of major academic teams. Figure 3B shows that flipped classroom was frequently cited in the 2020 online learning research. Some major terms used during 2021 included online teaching, surgical education, online learning experience, anatomy education, mental health, dental education, and cognitive load theory. The academics began to pay attention to online teaching and learning mode for postgraduate education in 2022. It is clear by now that online learning has changed from the initial focus on just a certain learning form (flipped curriculum) to the entire medical education, which shows the evolution of research in the 3 years since the COVID-19 outbreak.

#### 3.3.1. Online learning practice.

The first theme focused on online learning and education, including online teaching (cluster #0), flipped classroom (cluster #1), and online learning experience (cluster #3). Rose (2020) and Dost (2020) published the most valuable references, providing a solid research foundation for resolving the global pandemic. Some authors have also paid attention to underdeveloped countries that lack digital infrastructure and where students have poor e-learning capabilities^[14]^ so as to prevent e-learning from becoming another propeller of the educational gap between rich and poor countries. For example, Al-Balas (2020)^[15]^ discussed the experience of Jordan, while Khalil and Rehana (2020)^[16]^ explored that of Saudi Arabia.

#### 3.3.2. Learning theory and model.

Various theories and models related to pedagogy, psychology, and organizational behavior were employed to analyze the online learning behavior and motivation in the aftermath of the outbreak of the pandemic (Hadie Rajeh, 2021),^[17]^ including self-determination theory, cognitive load theory (cluster #7),^[18]^ and planned behavior theory. Other models, such as structural equation modeling (Almarzouqi, 2022) and expectation-confirmation model (Cheng, 2020), were used to assess user satisfaction and willingness to continue online learning.^[19]^

#### 3.3.3. Medical disciplines.

Some medical disciplines (cluster #2, cluster #4, and cluster #6) are attracting a wider audience within the online learning community due to their inherent complexity, particularly in subjects such as surgery, dentistry, and anatomy. Longhurst (2020)^[20]^ and Pather (2020) researched anatomy education, the most difficult subject among all medical education disciplines, Wilcha (2020) and Dedeilia (2020) reported on surgical education, while Amir (2020) and Schlenz (2020) engaged in dental education. These academic groups adapted the traditional, synchronous course to the online learning mode. In addition, some advanced technologies and tools, such as virtual auscultation,^[21]^ augmented reality,^[22]^ and simulation, were applied to these disciplines and desirable results were obtained.

#### 3.3.4. Mental health.

Mental health, the third theme in online learning practice (cluster #5) with 36 nodes, such as depression, stress, and anxiety, was reported on by Cao (2020) and Hodges (2021). The COVID-19 pandemic exposed the need to address mental health issues of medical students for a seamless adoption of e-learning. Some strategies have been proposed to address the factors that damage the mental health of medical students in online learning.^[23]^

#### 3.3.5. Lifelong learning.

The final theme (cluster #7) is postgraduate medical education with 14 nodes. According to Papapanou (2022), the quality of postgraduate medical education can be improved through online learning.^[24]^ Various online medical courses have been designed to meet the learning needs of students at all stages of medical education. At the beginning of the outbreak, undergraduate medical education focused initially on continuing the education (namely, continuing professional development). Later, postgraduate medical education also attracted the attention of both academic researchers and practitioners.

### 3.4. Keyword co-occurrence analysis

**Figure 4.**
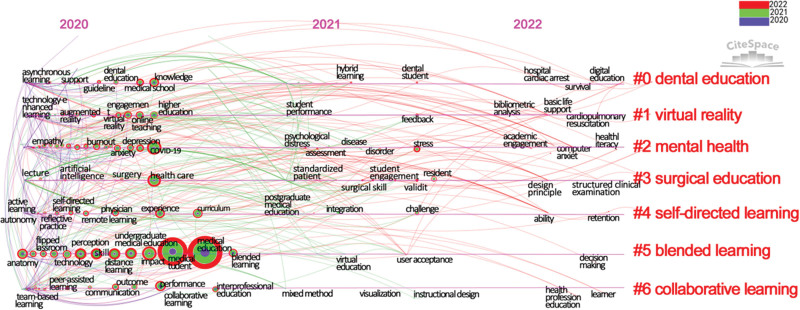
Keyword timeline visualization for online learning.

The keyword co-occurrence results did not show any burst keyword or keywords with a centrality of > 0.1. This result indicates that there is no obvious research center and core hotspot in the online learning field. Instead, there are decentralized themes and tendencies. Seven clusters were identified with the keyword timeline view by Cite Space (6.1.R3), including dental education, virtual reality, mental health, surgical education, self-directed learning, blended learning, and collaborative learning. According to the timeline view (Fig. 4) and the co-occurrence of keywords (Table 3), along with the number of chronological documents, the research oriented toward online learning during the COVID-19 pandemic can be roughly divided into the following 3 stages. Table 3 shows the top 10% keywords used in the period spanning from 2020 to 2022. Notably, 198 keywords appeared for the first time in 2020, which included almost all of the research on online learning to date. In addition, 154 keywords were noticed in 2021 with a lower word frequency and centrality, while only 53 new keywords appeared in 2022. Most of the high-frequency keywords appeared in the first year of the outbreak. Their frequency and centrality tended to reduce in the following years. However, this does not mean that the academics have lost interest in this field. We were pleased to see that these studies are moving toward a more refined and in-depth direction over time.

**Table 3:** Top high-frequency keywords in online learning from 2020 to 2022.

Year	Count	Centrality	Keywords	Year	Count	Centrality	Keywords
2020	775	0.01	medical education	2021	42	0.01	stress
	605	0.03	medical student		25	0.02	resident
	175	0	online learning		23	0.02	challenge
	155	0	covid-19		21	0.01	feedback
	145	0.01	impact		17	0.03	faculty development
	143	0.02	health care		16	0.02	perspective
	111	0	undergraduate medical education		15	0.02	validity
	104	0	distance learning		14	0.01	virtual education
	98	0.01	performance		13	0.01	student engagement
	98	0.05	skill		13	0.02	dental student
	95	0.04	perception		12	0.01	adolescent
	88	0.03	curriculum		11	0.01	self-efficacy
	86	0.02	anatomy		11	0.01	nurse
	76	0.01	online education		11	0.02	disorder
	73	0.01	technology		11	0.02	integration
	67	0.01	knowledge	2022	7	0	retention
	66	0.01	mental health		6	0	cardiopulmonary resuscitation
	62	0.02	experience		6	0.1	structured clinical examination
	61	0	blended learning		5	0	neurosurgery education
	60	0.01	medical school		5	0	digital education

#### 3.4.1. Rapid development stage.

As the largest distance-learning practice in the history of human civilization, online learning rapidly developed in 2020, accompanied by the weakening of the COVID-19 pandemic. However, note that the top 4 keywords should not be analyzed because they were search strategy terms. Health care, impact, and perception were identified as the most frequently cited keywords in this period. These keywords demonstrate the efforts made by the medical education community to ensure the effectiveness of online learning during the lockdown period. Some studies developed appropriate policies and initiatives to promote the effectiveness of e-learning.^[25]^ To achieve substantial equivalence, the research in 2020 mainly focused on the effects and factors of online learning.

#### 3.4.2. Flourish stage.

The online learning study method really flourished in 2021 when the COVID-19 pandemic maintained a stable deterioration trend. Compared with 2020, the keyword frequency and centrality greatly decreased in 2021. Most of the publications published in 2021 exhibited an increasing keyword and a decreasing frequency for keywords. The reduction in frequency indicates that the scholars tended to calm down after a rapid pace of research in 2020 and focus more on improving the depth and breadth of their research. The high-frequency keywords in 2021 mainly included stress, resident, challenge, feedback, faculty development, perspective, and validity. The research direction also changed from an online learning guarantee to quality improvement. Other studies demonstrated the possibility of changing the learning path from real teaching to virtual online learning during the pandemic.^[26]^

#### 3.4.3. Stable stage.

After 2 years of rapid development, online learning has now entered a stable stage with a more mature theory and more diverse methods. The frequency of keywords, such as retention, cardiopulmonary resuscitation, neurosurgery, and structured clinical examination, also gradually reduced. This result indicated that the attention to some important topics within the existing research gradually reduced and replaced by various contextualized situational micro-topics, which is a manifestation of more in-depth research. The new stage focused on topics such as self-regulated learning, advanced technology application, and teaching models. Moreover, scholars now paid more attention to exploring a hybrid teaching method by combining both traditional classroom and online learning. They are also committed to transforming and retaining online learning results and experiences, even in the post-pandemic era.

This comprehensive analysis underscores the nuanced progression of research focus, which traverses a spectrum from immediate pandemic-driven responses to the refinement of quality and efficacy, culminating in the adoption of advanced blended strategies and interdisciplinary perspectives.

## 4. Discussion

Medical education can be divided into pre- and post-COVID-19 pandemic periods.^[27]^ This study attempts to identify changes in online medical education during these periods and whether such changes can adequately serve the training needs of future medical practitioners.

### 4.1. Autonomous online learning

Spatiotemporal asynchrony induced by the COVID-19 pandemic requires a higher autonomy of medical students.^[28]^ The theory of autonomous learning, such as self-directed learning, self-regulated learning, self-reflective learning, and others, were realized during this unprecedented large-scale online education practice and fundamentally changed the learning ideology. In an online teaching and learning context, knowledge is transferred from passive infusion to positive acquisition and promotes the application of knowledge in clinical practice through self-reflection. Although this change took place in a relatively short period of time, it was sufficient to produce an active learning “habitus” of the practitioner (learners and educators) even after the pandemic. However, only limited scholarly literature explores the lifelong self-directed learning by medical students and physicians, including undergraduate medical education, post-graduation medical education, and continuing medical education.

### 4.2. Diversified online learning technology

A wide range of methods, tools, and modalities have been integrated into online learning. At the same time, we need to focus on how the research on medical education is pursuing these advanced technologies. For example, a great deal of research is being conducted to explore the use and effectiveness of various technologies such as augmented reality, digital simulations, virtual and remote laboratories, and synchronous/asynchronous learning tools. Besides, many attempts have been made to apply some of the advanced topics in online learning such as artificial intelligence, cloud computing, blockchain, knowledge engineering, and software engineering method. However, the use of these technologies is putting increasing demands on the online learning ability of students, including their ability to apply information technology, self-control, and pressure regulation. For policymakers, researchers, and practitioners, it is more important to focus on the technology in innovative pedagogy than on technology solely.

### 4.3. In-depth integration of blended-learning modes

The blended-learning mode involving online and offline in-depth integration is gaining increasing attention in the post-pandemic era. Note that blended or hybrid learning does not simply represent Online-Merge-Offline activities. It can also not be considered a return to pre-pandemic blended learning. As a deep integration, blended leaning draws on the experiences and practices observed during the pandemic and provides students with a ubiquitous, personalized, and targeted learning support.^[29]^ In addition, efforts are being made to improve the quality of blended-learning strategies. Some studies have attempted to explore the application of the blended teaching model based on small private online courses, MOOC,^[30]^ problem-based learning, team-based learning,^[31]^ and case-based learning. Finally, academia believes that the evaluation criteria need to be redefined to achieve multidimensional evaluation.^[32]^

### 4.4. Cooperative online learning strategies

Our study showed that countries, institutions, and authors are more deeply interconnected than we knew. This interconnection depends on a network of cooperation and exchanges. The Internet provides an opportunity for underdeveloped countries to acquire medical educational resources from advanced countries. That is why these countries are so keen to participate in online learning research. After all, one of the purposes of international exchanges and cooperation is to promote educational equity. An additional analysis – Journal Overlay Map – demonstrated that information science, engineering, psychology, and sociology are involved in online education research, exhibiting an advantage over medical and educational disciplines. Online education was able to overcome the adverse effects of the pandemic because all educational organizations strive to produce and share knowledge. In the post-pandemic era, the international first-class curriculum system will be based on the cooperation and division of labor among countries and institutions. Medical schools around the world will carry out extensive cooperation to train transnational medical and public health students, decision-makers, and clinicians. The form of cooperation has also changed from a single discipline to an interdisciplinary and transdisciplinary direction.^[33]^

## 5. Conclusion

This study undertook a rigorous bibliometric analysis along with visualization techniques to comprehensively investigate the landscape of online learning research within the context of medical education during and subsequent to the COVID-19 pandemic. Through an analysis of publications, co-cited references, co-occurring keywords, and collaboration networks, the study shed light on research trends, collaborative networks, and principal focus areas.

The comprehensive bibliometric analysis conducted herein facilitated an in-depth comprehension of global scholarly engagement in addressing a diverse array of issues related to online learning in response to the pandemic. The study underscored that the academic response to this global challenge was characterized by extensive international collaboration, with a prominent role played by the United States and notable contributions originating from Chinese authors. The co-cited references provided insight into the intellectual continuum between preceding and evolving research endeavors in the realm of online learning, predominantly accentuating practical implementations and experiences, widely embraced theories and models, curriculum redesign across varied disciplines, psychological implications stemming from the pandemic, and enduring challenges of lifelong learning. Through a meticulous examination of keyword co-occurrence analysis, it became evident that the trajectory of research evolution traversed distinct 3 stages, mirroring a progression from immediate pandemic-driven responses, to the refinement of quality and efficacy, and culminating in the adoption of more advanced strategies and interdisciplinary perspectives. Remarkably, all subject domains, encompassing intricate fields such as anatomy, dentistry, and surgery, successfully navigated the intricacies of online learning, thereby reinforcing the robustness of research focal points and emerging trends.

The culmination of these findings, in conjunction with nuanced insights derived from the co-occurrence analysis of keywords and timeline visualization, elucidates a profound evolution trajectory for online learning. This trajectory is poised to embrace heightened learner autonomy, intensified integration of advanced technologies and methodologies, and a more profound synthesis of blended learning methodologies. Furthermore, the trajectory envisions a heightened emphasis on extensive interdisciplinary and transdisciplinary cooperation, indicative of a departure from the constraints of singular disciplines. As the global landscape transitions beyond the pandemic, the trajectory augurs an intricate interplay of interconnectedness, innovation, and adaptability in the future of online learning. This transformative shift in online learning has the potential to engender profound transformations in medical education, equipping both learners and educators with the resilience and agility requisite for navigating the dynamic terrain of modern healthcare education.^[34]^

In conclusion, this study furnishes a robust scholarly foundation for anticipating and navigating the unfolding paradigm of online learning for medical students worldwide in the post-COVID-19 era. The amalgamation of bibliometric insights, co-cited references, and keyword co-occurrence analyses collectively provides a comprehensive compass to steer future research endeavors and policy formulations, ensuring the continued evolution of medical education in harmony with the shifting currents of technology, collaboration, and pedagogical innovation.

## 6. Limitations

This study has several limitations. Our data is incomplete and is only exported from the WoSCC. The second limitation is related to bias. Although the WoSCC is the most frequently used database in the scientific metric research, some different expressions with the same meaning were identified and integrated manually. These problems may be resolved in the future with software updates.

## Acknowledgments

We thank the university library for assistance with literature retrieval. We also thank https://www.fcedit.com/en/index.aspx for language editing service.

## Author contributions

**Conceptualization:** Haihun Zhai.

**Data curation:** Pei Zhang, Xiuyuan Li, Ying Pan.

**Formal analysis:** Pei Zhang, Xiuyuan Li, Ying Pan, Tian Li.

**Funding acquisition:** Haihun Zhai.

**Investigation:** Xiuyuan Li, Ying Pan.

**Methodology:** Pei Zhang, Haihun Zhai.

**Resources:** Haihun Zhai.

**Software:** Pei Zhang, Tian Li.

**Validation:** Haihun Zhai.

**Visualization:** Pei Zhang.

**Writing** – **original draft:** Pei Zhang, Xiuyuan Li.

**Writing** – **review & editing:** Pei Zhang, Ying Pan.
